# Supplementary figures and images for: Development of a cell-free screening assay for the identification of direct PERK activators
Source: PLoS One. 2023 May 18;18(5):e0283943. doi: 10.1371/journal.pone.0283943 (PMC10194977; doi:10.1371/journal.pone.0283943)

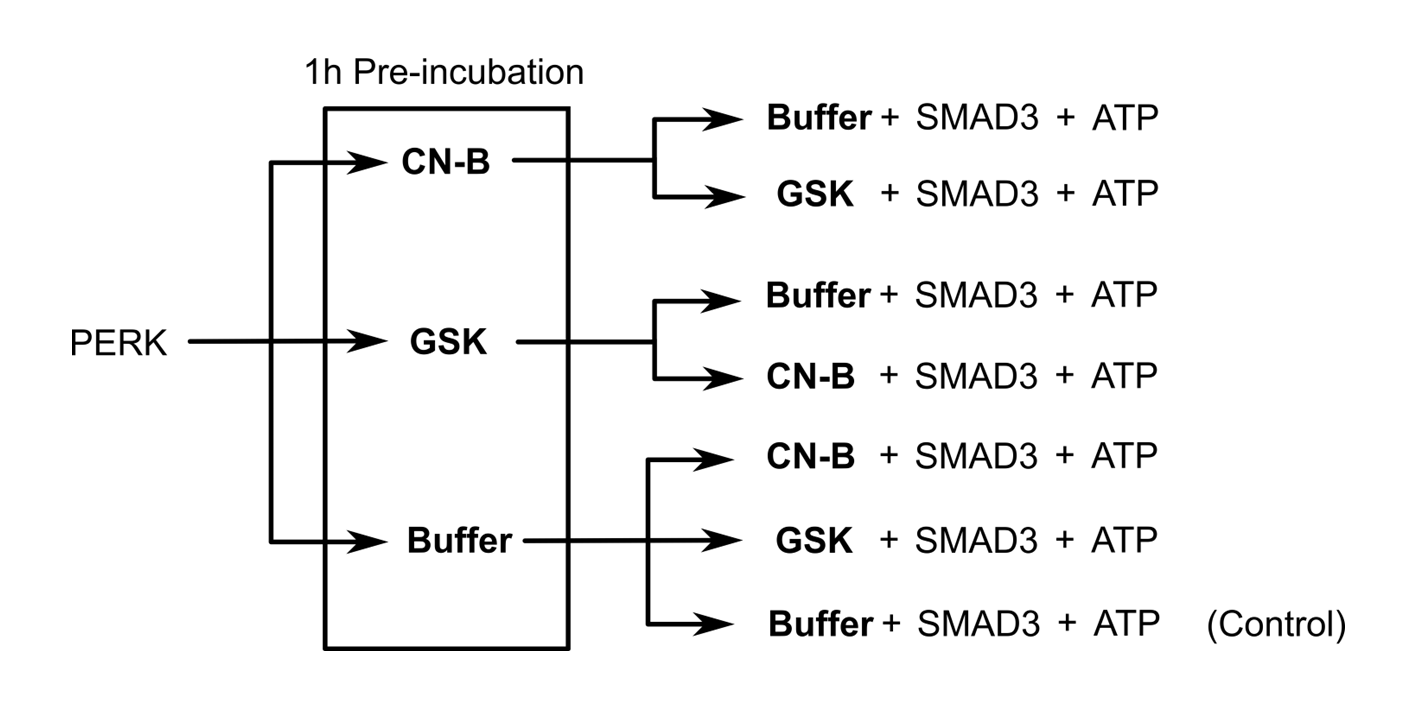

Supplement: S1 Fig — PERK was pre-incubated with CN-B, GSK2606414 or buffer. After the pre-incubation, PERK was alternatively ‘rescue’ with CN-B, GSK2606414 or buffer, as indicated in the diagram. Finally, SMAD3 and ATP were added to start the kinase reaction. The ATP consumption was measured as detailed in the materials and methods section. (TIF) [file pone.0283943.s001.tif]

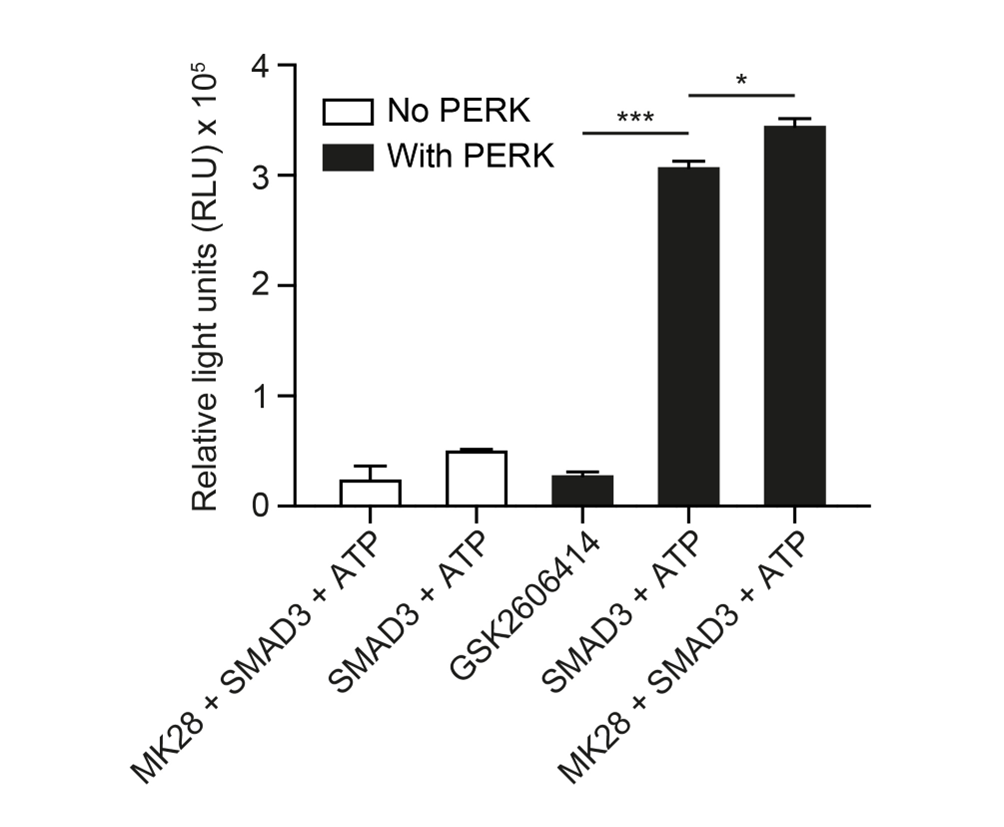

Supplement: S2 Fig — MK-28 effect on ATP consumption was measured in absence and presence of PERK and expressed as RLU. Data are mean ± SEM from 3 technical replicates. Statistical analysis was Student’s t-test. *p < 0.05 and ***p < 0.001. (TIF) [file pone.0283943.s002.tif]
