# Supplementary material for: Development of a cell-free screening assay for the identification of direct PERK activators
Source: PLoS One. 2023 May 18;18(5):e0283943. doi: 10.1371/journal.pone.0283943 (PMC10194977; doi:10.1371/journal.pone.0283943)
Supplement: Attachment — Submitted filename: Response to Reviewers.docx [file pone.0283943.s003.docx]

**Reviewer´s comments:**

**Comment 1:***Fig. 2B. There is practically no increase in the signal obtained in the assay using the natural PERK substrates. Even the increase with the unnatural substrate SMAD3 is very small, although in this case it gave statistical significance. These results point to a lack of robustness of the assay and put in doubt its validity to measure the natural activity of PERK. Although it might still be useful to screen for PERK activators, these limitations of the study should be discussed in the text.*

**Answer:** Thank you for the comment. As stated by the reviewer, the increase in PERK activity by SMAD3 was small, although statistically significant. However, in our view, this is not necessarily suggestive for a lack of robustness or validity. The aim of this particular experiment was to simply understand if SMAD3, routinely used in the radioactive assay protocol setup by the manufacturer SignalChem, is a suitable protein substrate in our setup. For this reason, we compared PERK´s ATP consumption in presence of SMAD3, or in presence of PERK´s natural substrates (eIF2α and NRF2). Although PERK is capable of autophosphorylation, leading to ATP consumption even in the absence of a protein substrate, an increase in ATP consumption is expected when a substrate protein is present. Indeed, as shown in Fig 2B, we observed an increase in ATP consumption when PERK reacted in presence of any of the tested substrate protein, suggesting that both SMAD3 and PERK´s natural substrates are working as phosphorylation-accepting proteins. Moreover, the increase in ATP consumption (suggestive of a gain in PERK activity) was higher and statistically significant for SMAD3, thus reinforcing the cost-efficiency of SMAD3 compared to PERK´s natural substrates.

The further experiments, such as PERK modulation with GSK2606414 or calcineurin-B (Fig 2D), and the measurement of an activating EC50-value (Fig 3C) provide evidence that the developed assay is robust and can be used to screen for PERK activators. Nevertheless, we acknowledge the reviewer’s comment and integrated the mentioned aspect in our manuscript to point out limitations of our study in line 510-517, also concerning the use of SMAD3.

**Comment 2:** *It is unclear why SMAD3 was chosen as a substrate, as it is not a natural substrate of PERK.*

Answer: Thank you for this comment. SMAD3 is used in routine analysis to measure PERK’s specific activity with a radioactive assay protocol setup by the manufacturer SignalChem. It is a cost-efficient alternative for PERK’s natural substrates, such as eIF2α or NRF2, which are both very expensive. Because we developed our assay for a future application in high-throughput screening (HTS) using large amounts of proteins, this is an important point to consider. To make it this point clear, we modified the sentence in line 333-336.

**Comment 3:** *The use of the unnatural substrate SMAD3, of only the catalytic domain of PERK and of a reaction at room temperature instead of 37C, takes the developed assay away from the natural PERK biology. While it might still be useful to screen for PERK activators, this should be commented and discussed as a limitation of the study.*

Answer: Thank you for the comment. We added this point to line 509-517 and state more clearly possible limitations of our work.

**Comment 4:** *The statement in the Abstract about the “…non-availability of direct PERK activators…” as well as at the beginning of the Discussion and in other parts of the text is misleading, as MK-28 has been shown to be a direct PERK activator in a cell-free assay (Ganz el al, 2019) and confirmed now in this manuscript. These statements should be removed or changed.*

Answer: We changed the manuscript accordingly. To avoid misunderstandings, we changed the statement in the Abstract (line 24) from “non-availability of direct PERK activators” to “a shortage of available direct PERK activators”. In other formulation we state e.g., “lack of direct PERK activators” (line 83) or “lack of available compounds that directly activate PERK” (line 455). We believe that the term “lack” states correctly that there is not a complete absence but rather very limited availability.

**Comment 5:** *Fig. 1A, and lines 258-9. “…cellular screening assays may identify compounds that activate the PERK pathway in an unspecific manner…” Although this could be true in general, there are ways to test the specificity to PERK, by measuring the effect of the compounds in PERK knockout cells compared to WT cells. This should be added/ commented in the figure and in the text.*

Answer: Thank you for this point. We added this aspect to our manuscript in line 263, and by changing the Fig 1A legend to “by measuring distinct activated target proteins or by comparing treated PERK knockout cells with wild-type cells other pathways of the UPR can be excluded” (line 274-275).

**Comment 6:** *Lines 259-261 “Contrariwise, cell-free screening systems, by restricting the reaction to the target protein, allow the identification of test compounds that specifically and directly interact with the PERK kinase (Fig 1B).” This statement gives the impression that direct interaction is measured in the manuscript, which is not the case. Direct action on PERK can be inferred but direct interaction has not been measured. The statement should be removed or moderated.*

Answer: We agree to the reviewer’s comment and carried out the following changes to the manuscript for clarification:

- line 269 from “directly interact with the PERK kinase” to “directly modulate PERK kinase activity”
- Fig 1B from “direct interaction” to “direct modulation”
- Fig 1B legend, line 278 from “directly interact with PERK” to “directly modulate PERK kinase activity”
- line 465 from “direct target interaction” to “direct modulation”
- line 469 from “directly interact with the target” to “directly modulate the target”.

**Comment 7:** *Line 277 “At present, there is no commercial PERK activator assay available.” and line 458 “…there is no cell-free screening kit for PERK activators obtainable…” However, there are companies that have cell-free PERK activator assays, and one can make use of their services (see e.g. in Ganz el al 2019). This should be commented in the text.*

Answer: Presently, no commercial cell-free PERK activator assay is available. However, kinase inhibitor assays can be commercially obtained. Alternatively, full kinase panels for measurement of kinase activity are available at specialized companies. Originally, *Ganz et al* developed MK28 from a compound termed “A4”, previously found in a virtual library screening by *Wang et al* (2010). After chemical modification, *Ganz et al* found MK28 to be more potent, and confirmed its specificity to activate PERK on a full kinase panel analysis, carried out by a company. But in conclusion, *Ganz et al.* didn’t use a commercially available PERK activator assay.

We do acknowledge that there are alternative methodologies, such as radioactive assays to screen for kinase modulators, as we referred in line 470, but they are intended to screen for inhibitors. We also pointed out that these methodologies are not present in all laboratorial facilities and that our developed assay has the advantage to use non-radioactive conditions with comparable sensitivity.

**Comment 8:** *Line 281 “…protein substrate SMAD3 from the original radioactive protocol and applying it…”. It is unclear to what original radioactive protocol the authors are referring to.*

Answer: With this phrase, we refer to the radioactive protocol which is used by the manufacturer of PERK (SignalChem) to determine the specific activity of PERK as part of a routine protein quality analysis. To clarify this, we made changes to line 92-94 and line 290-291.

**Comment 9:** *Lines 410-412 “Our findings strongly suggest that calcineurin-B place of action differs from the inhibitor GSK2606414 and activates PERK by a mechanism independent of the active site which is blocked by GSK2606414.” The authors should speculate in the text what the mechanism of activation could be, based on previous studies on the mechanisms of PERK activation.*

Answer: Based on previous studies reporting PERK activation by calcineurin (Bollo et al, 2010; Chen et al, 2016), we tested and confirmed that calcineurin-B promotes PERK activity in our assay. We did not investigate the mechanism behind this activation, as it was not the scope of our project. However, based on our results, we speculated a possible mechanism of action, in lines 493: “calcineurin-B might increase PERK activity not by directly interacting with PERK’s catalytic site, but by favoring protein-protein interactions between PERK and its protein substrate”. We stated, that this speculation comes from the fact that “we observed that calcineurin-B does not favor PERK autophosphorylation and only activates PERK in the presence of a substrate protein” (line 490-491), together with “evidences of a weak PERK-calcineurin B-interaction reported by others” (line 492). Indeed, as we also noticed (line 496) “improving stability of kinase-substrate interaction, by targeting structural features prone to disruption, is a possible mechanism of action of kinase activators” (Lopez et al, 2020).

To address the reviewer´s comment, we made additional changes to line 495.

**Comment 10:** *Although the English is good in general, there are some mistakes in the grammar. (for example in line 373 “…when PERK reacted in the presence of calcineurin-B was neither not due to a direct…”). The text should be edited by a native English speaker.*

Answer: Thank you for the comment. We corrected the mentioned error accordingly in line 385. The entire manuscript was doublechecked by a native English speaker, who we now include in the acknowledge section.
